# Impact Analysis of Photoperiodic Disorder on the Eyestalk of Chinese Mitten Crab (*Eriocheir sinensis*) through High-Throughput Sequencing Technology

**DOI:** 10.3390/life14020209

**Published:** 2024-01-31

**Authors:** Baoli Zhang, Yuqiao Chai, Yingkai Xu, Ziwei Huang, Xueqing Hu, Yingdong Li

**Affiliations:** Key Laboratory of Livestock Infectious Diseases in Northeast China, Ministry of Education, College of Animal Science and Veterinary Medicine, Shenyang Agricultural University, Shenyang 110866, China

**Keywords:** *Eriocheir sinensis*, eyestalk, photoperiod, transcriptome

## Abstract

Light is an indispensable factor in the healthy growth of living organisms, and alterations in the photoperiod can have consequences for body homeostasis. The eyestalk is a photosensitive organ that secretes various hormones to regulate the Chinese mitten crab (*Eriocheir sinensis*). However, the photoperiod-dependent eyestalk patterns of gene expression that may underlie changes in body homeostasis are unknown. In this study, we investigated the molecular mechanisms involved in eyestalk transcriptomic responses in *E. sinensis* under different photoperiod regimes on days 2, 4, and 6. The photoperiods tested were 12, 24, and 0 h light/day. In total, we obtained 110, 958, 348 clean datasets and detected 1809 differentially expressed genes (DEGs). Genes involved in the crustacean hyperglycemic hormone superfamily and juvenile hormones were observed, which play important roles in gonadal development, growth, and immunity in *E. sinensis* and may also be involved in photoperiod adaptation. In addition, the MAPK signaling pathway was the only signaling pathway identified in the continuous light group but was absent in the continuous darkness group. We suggest that the MAPK pathway is highly responsive to light input during the subjective night and insensitive to light during the middle of the subjective day. These results provide insight into the molecular mechanisms underlying the effects of photoperiod on the immune regulation of *E. sinensis*.

## 1. Introduction

Circadian rhythms have been extensively explored in animal physiology, revealing their pivotal role in governing fundamental processes such as sleep–wake cycles, dietary patterns, metabolism, immune responses, and hormone regulation [1,2,3]. Animals exhibit distinct activity–rest rhythms that synchronize with the day–night cycle, enabling them to finely regulate metabolic functions and adapt to environmental changes based on circadian cues [4,5]. The photoperiod serves as a key cue for animals to modulate their life activities. Fish growth and gonadal development are influenced by the photoperiod, with long-day manipulation applied for healthy growth and early gonadal maturation in ornamental fish [6,7]. Crustaceans, such as crayfish, are susceptible to light signals that affect molting frequency, growth, and reproductive efficiency [8,9].

The eyestalk in crustaceans serves as the central hub of the circadian system, orchestrating key physiological events such as molting, growth, and reproduction [10]. Recent studies highlight the involvement of eyestalk neuroendocrine hormones in circadian rhythms [11,12]. Among the neuroendocrine factors regulating crustacean reproduction, molt-inhibiting hormone (MIH), gonad/vitellogenesis-inhibiting hormone (GIH/VIH), melatonin, crustacean hyperglycemic hormone (CHH), and mandibular organ-inhibiting hormone (MOIH) are crucial for molting, growth, and gonadal development [13,14,15]. Systematic exploration of the eyestalk provides a unique opportunity to unveil the molecular mechanisms underlying circadian rhythms, decipher the signaling pathways governing essential physiological processes, and understand how these processes interact with environmental changes.

Chinese mitten crab (*Eriocheir sinensis*) is farmed in more than ten coastal cities and inland provinces and cities and is a unique and famous aquatic product in China. In 2022, the farming area of Chinese mitten crabs in China totaled 12.6 million mu, with a national production of 782,200 t and a demand of 776,900 t [16]. Chinese mitten crab is economically significant because of its substantial market value and widespread distribution [17]. Surprisingly, there is a gap in research on how alterations in the light cycle affect eyestalk function in *E. sinensis*. Thus, research on optimizing photoperiod conditions could potentially enhance the growth rate, reproduction, and overall well-being of captive populations. Therefore, addressing the dual aspects of the ecological and economic significance of photoperiod changes in Chinese mitten crabs, with a specific focus on eyestalk responses, will substantially contribute to the scientific understanding of this species and inform strategies for its sustainable management and cultivation.

## 2. Materials and Methods

### 2.1. Ethics Statement

The Chinese mitten crab (*Eriocheir sinensis*) used in our study is not an endangered or protected species; it is a common edible crab in China and no permit is required to catch it. All the experiments were conducted in accordance with the guidelines for scientific purposes, animal care, and use established by the Animal Ethics Committee of Shenyang Agricultural University.

### 2.2. Experimental Design and Sampling

**Figure 1:**
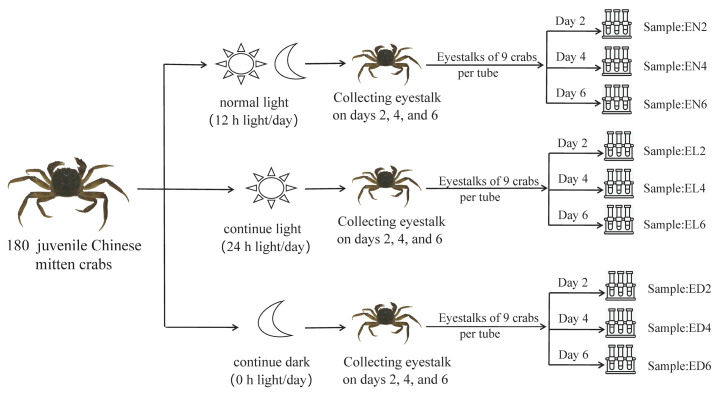
Schematic showing the experimental design and sampling procedures for the transcriptome analysis of *Eriocheir sinensis*. Notes: EN2 (normal light for 2 days), EL2 (continuous light for 2 days), ED2 (continuous darkness for 2 days), EN4 (normal light for 4 days), HL4 (continuous light for 4 days), ED4 (continuous darkness for 4 days), EN6 (normal light for 6 days), EL6 (continuous light for 6 days), and ED6 (continuous darkness for 6 days).

The juvenile Chinese mitten crabs (body weight: 8.15 ± 2.35 g) were acquired from Panjin Guanghe Crab Industry Co., Ltd., Panjin City, Liaoning Province, China, and acclimated in an indoor circulating water system at the aquatic laboratory of Shenyang Agricultural University. The experimental setup is illustrated in Figure 1. After a two-week acclimation, a total of 180 juvenile crabs were randomly assigned to three groups, each housed in three tanks (density: 20 crabs/tank) measuring 50 cm × 30 cm × 20 cm (length × width × depth), with a water depth of 5 cm. The temperature was maintained at 15 °C, and crabs were fed pelleted dry food at a daily rate of 1% of their body weight. 

Three experimental groups were exposed to different light conditions: normal light (12 h light/day, serving as the control), continuous light (24 h light/day), and continuous darkness (0 h light/day). The trial spanned six days, and eyestalks were collected from crabs at 12:00 on days two, four, and six under each photoperiodic condition. Crabs in the continuous dark group were sampled in the dark. All collected samples were stored in liquid nitrogen. The sample labels were as follows: EN2, EL2, ED2, EN4, EL4, ED4, EN6, EL6, and ED6.

### 2.3. RNA Isolation and RNA-Seq Library Preparation

Eyestalks were frozen with liquid nitrogen and pulverized in a grinding bowl, and total RNA was extracted using a FastPure^®^ Cell/Tissue Total RNA Isolation Kit V2 (Vazymc, Nanjing, China) according to the manufacturer’s instructions. Three replicates were performed for each treatment group. The integrity of the extracted RNA was monitored using 1% agarose gels. The purity and concentration of RNA samples were assessed using a NanoDrop spectrophotometer (Thermo Scientific, Shanghai, China). Transcriptome sequencing was performed using an Illumina Truseq 2000 sequencing platform by Shanghai Personal Biotechnology Co., Ltd. Shanghai Biotechnology Corporation (Shanghai, China) performed the transcriptome sequencing of the target samples.

### 2.4. Transcriptome Data Processing and Analysis

Cutadapt (v1.15) software was used to filter the sequencing data to obtain high-quality sequences (clean data) for further analysis. The reference genome and gene annotation files were downloaded from the genome website (http://www.genedatabase.cn/esi_genome.html; accessed 6 December 2023). The filtered reads were mapped to the reference genome using HISAT2 v2.0.5. All transcripts were compared with the Swissprot, Nr, eggNOG, GO, and KEGG databases for functional annotation using the Basic Local Alignment Search Tool (BLAST, V5) (E-value < 1 × 10^−5^). We mapped all genes to terms in the Gene Ontology database and calculated the number of differentially enriched genes for each term. Using topGO to perform GO enrichment analysis on the differentially expressed genes, the *p*-value was calculated using the hypergeometric distribution method (the standard of significant enrichment was *p*-value < 0.05). Cluster Profiler (3.4.4) software was used to carry out enrichment analysis of the KEGG pathway of differential genes, focusing on the significantly enriched pathway with a *p*-value < 0.05.

### 2.5. Differential Gene Expression Analysis

We used HTSeq (0.9.1) statistics to compare the read count values for each gene with the original expression of the gene, and then used FPKM to standardize the expression. The difference in gene expression was analyzed using DESeq (1.30.0) under screened conditions as follows: expression difference multiple |log2FoldChange| > 1, significant *p*-value < 0.05.

### 2.6. Quantitative Real-Time Polymerase Chain Reaction (qRT-PCR)

To validate the RNA-Seq results, six genes were subjected to RT-qPCR analysis. Primers for PCR were designed using Primer Premier software (version 6.0) (Table 1). *β-Actin* served as the internal control, and each sample underwent triplicate analysis. PCR was conducted with Hiscript^®^ III RT SuperMix for qPCR (+gDNA wiper) (Vazymc, China) in a 20 μL reaction system. RT-qPCR was performed in a total volume of 20 mL, with cycling conditions of 95 °C for 30 s, followed by 40 cycles of 95 °C for 10 s, 60 °C for 15 s, and finally, 95 °C for 10 s, 65 °C for 60 s, and 97 °C for 1 s. Dissociation curve analysis was performed after each qPCR. The 2^−ΔΔCt^ method was employed to determine relative changes in gene expression levels. Results, presented as mean ± SD, underwent one-way analysis of variance (ANOVA) using SPSS 22.0, with *p* < 0.05 considered statistically significant.

## 3. Result

### 3.1. Transcriptome Sequence Assessment and Annotation

A total of 130,075,274 raw reads were obtained, with a Q30 exceeding 90.64% (Table S1). After stringent filtering, 110,958,348 high-quality clean reads were retained (Table S2). Annotations against major databases revealed 78,701; 50,358; 36,889; 51,563; and 67,040 unigenes mapped to NR, GO, KEGG, SwissProt, and eggNOG, respectively (Table S3).

### 3.2. Differential Expression Analysis

**Figure 2:**
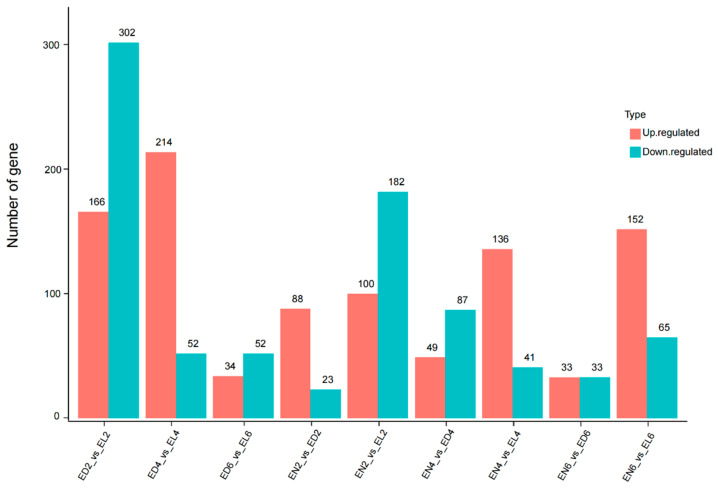
Bar graph of gene expression between groups. The abscissa indicates the comparison group for difference analysis. The ordinate indicates the number of differential genes. Pink and light blue represent upregulated and downregulated genes, respectively.

DESeq analysis of eyestalks at different photoperiods identified 25,489 annotated genes, of which 1809 were differentially expressed genes (DEGs) (Figure 2). DEGs related to molting, growth, and immunity were selectively screened (Table 2). Molting-related DEGs included *cuticle 7* and *cuticle proteins* in the continuous darkness groups (EN2 vs. ED2 and EN4 vs. ED4). Immune-related DEGs included *crustin 4*, *cathepsin L 1-like*, *perlucin 5*, *cathepsin H*, *c-type lectin*, *integrin alpha 4*, *cytochrome P450 CYP2*, *catalase*, *anti-lipopolysaccharide factor 2*, and *anti-lipopolysaccharide factor 3*. Interestingly, hormone-related genes showed no significant differential expression (Table 3). More DEGs were exhibited in the continuous light group than in the continuous darkness group, and the number of DEGs decreased with prolonged photoperiod exposure.

### 3.3. GO Functional Classification of Differentially Expressed Genes

**Figure 3:**
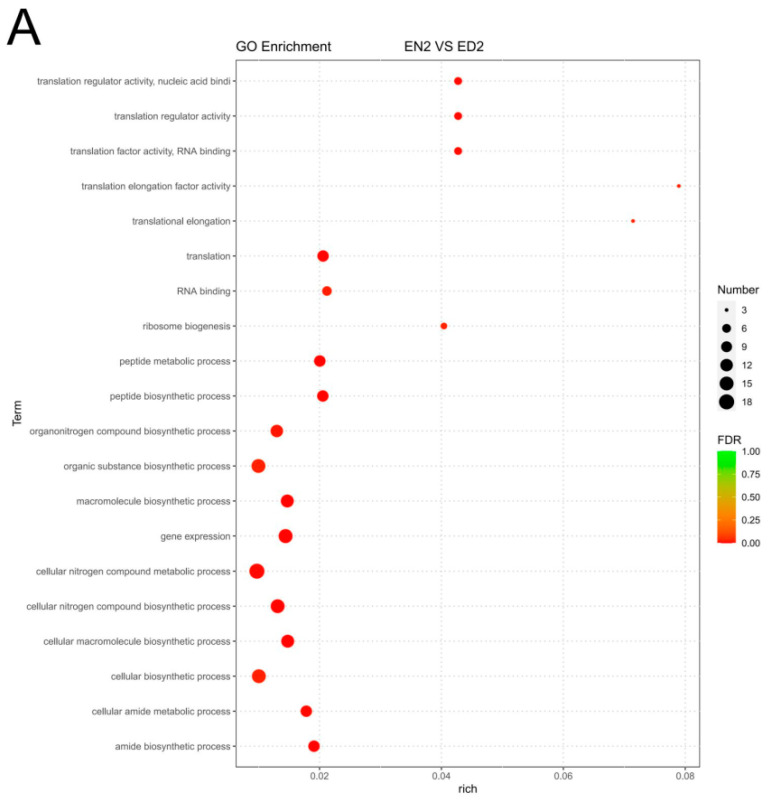

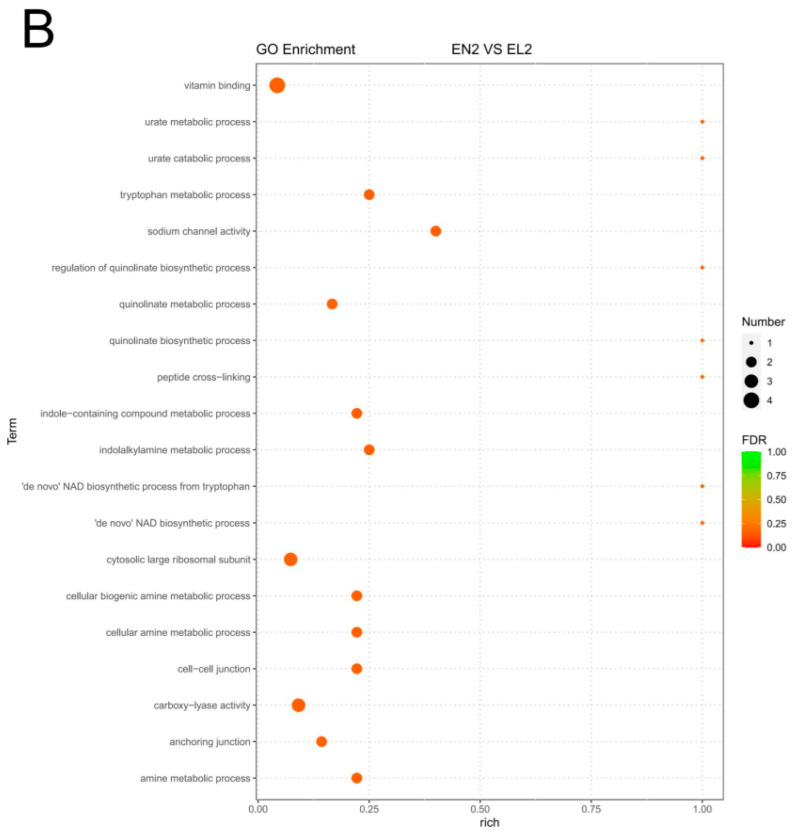
Gene Ontology (GO) classification showing DEGs for *Eriocheir sinensis* eyestalk in EN2 vs. ED2 (**A**), EN2 vs. EL2 (**B**).

GO analysis of DEGs highlighted the key biological processes, cellular components, and molecular functions affected by photoperiod. On day 2 of continuous darkness (EN2 vs. ED2), DEGs were enriched in terms of translation and RNA binding, whereas continuous light (EN2 vs. EL2) showed enrichment in sodium channel activity and vitamin binding (Figure 3A,B). On day 4, continuous darkness (EN4 vs. ED4) was associated with purine-containing compound catabolism, whereas continuous light (EN4 vs. EL4) resulted in organelle and intracellular enrichments (Figure S1). On day 6, continuous darkness (EN6 vs. ED6) revealed terms related to binding, nucleotide binding, and nucleoside phosphate binding. Continuous light (EN6 vs. EL6) enhanced the metabolic activity of genes involved in translation and cellular amide metabolic processes. Continuous darkness/light (ED6 vs. EL6) was linked to the plasma membrane and organic matter transport (Figure S2).

### 3.4. Significant Enrichment Analysis of KEGG Pathway

**Figure 4:**
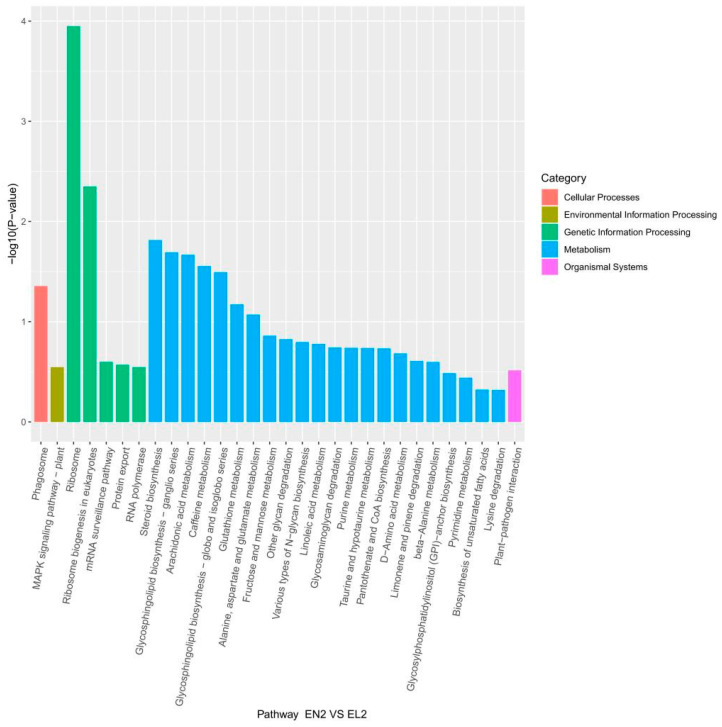
Classification statistics of KEGG metabolic pathways showing DEGs for *Eriocheir sinensis* eyestalk in EN2 vs. EL2.

To elucidate the effect of light exposure on these pathways, we conducted a KEGG pathway analysis of the DEGs. On day 2, DEGs were prominently enriched in gene information processing pathways, specifically ribosomes and ribosome biogenesis in eukaryotes (Figure S3). Notably, no signaling pathways were identified on day 2 of continuous darkness (EN2 vs. ED2), whereas the MAPK signaling pathway was observed on day 2 of continuous light (EN2 vs. EL2; Figure 4).

On day 4, phagosomes and ribosomes were the prominent pathways, of which metabolism was the most enriched (Figure S4). In the metabolism category, the citrate cycle (TCA cycle) was significantly upregulated in the continuous light group (EN4 vs. EL4), indicating enhanced metabolism (Figure S5). Other metabolic pathways, such as caffeine metabolism, nitrogen metabolism, terpenoid backbone biosynthesis, and galactose metabolism, were highlighted under continuous darkness (EN4 vs. ED4). The MAPK signaling pathway was exclusively found in the continuous light group, which was consistent with the results on day 2.

On day 6, the DEGs primarily acted through the phagosomal pathway in cellular processes (Figure S6). Continuous darkness (EN6 vs. ED6) resulted in fewer metabolism-related pathways than continuous light (EN6 vs. EL6), with only glutathione metabolism and five carbohydrate metabolism-related pathways (Figure S7B,C). Intriguingly, the MAPK signaling pathway reappeared as the sole signaling pathway under continuous light.

### 3.5. qRT-PCR Verification

**Figure 5:**
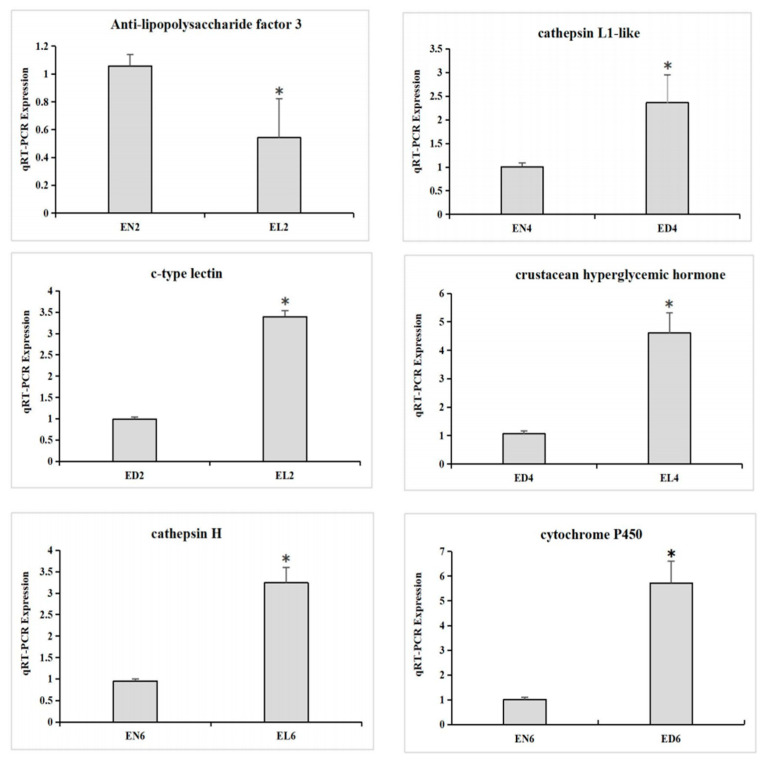
qRT-PCR verification of differentially expressed genes. “*” represents significant differences at *p* < 0.05.

To validate the RNA-Seq data, six DEGs with significant differences in expression—*anti-lipopolysaccharide factor 3*, *cathepsin L1-like*, *cathepsin H*, *crustacean hyperglycemic hormone*, *c-type lectin*, and *cytochrome P450*—were selected for qRT-PCR analysis. The expression levels of these DEGs were significantly correlated with the RNA-seq results (Figure 5).

## 4. Discussion

The necessity to synchronize genetic, physiological, and behavioral processes with diurnal shifts underscores the fundamental challenges on Earth [18]. Light is a crucial variable for aquatic organisms, especially those in natural and farmed ponds, and plays a pivotal role in orchestrating complex biological systems [19]. Our investigation delves into the neuroendocrine intricacies of the Chinese mitten crab, a unique, economically important crustacean, by employing next-generation sequencing to scrutinize the eyestalk under varying photoperiods, specifically after two, four, and six days.

Understanding the circadian rhythms governing feeding activity, metabolism, melatonin, and hyperglycemic hormones in crustaceans laid the foundation for our exploration [20,21,22]. Notably, disruptions in melatonin secretion have been observed in continuous light and dark environments, with the most pronounced changes occurring at 48 h of treatment, suggesting a critical time point for photoperiodic influence [12]. Consistent with these findings, the oscillation of cryptochrome abundance in crayfish brains exhibited a 24 h rhythm, with an 8 h delay after 24 and 72 h of darkness [23]. Consequently, this study aimed to decipher the pathways of Chinese mitten crab responses to photoperiodic alterations.

Previous studies in crustaceans have shown that the X-organ–sinus gland complex (XO-SG) in the eyestalk is an important neuroendocrine regulatory organ, with particularly superior expression of the crustacean hyperglycemic hormone superfamily [24]. The crustacean hyperglycemic hormone (CHH), molt-inhibiting hormone (MIH), gonad-inhibiting hormone (GIH), and mandibular organ-inhibiting hormone (MOIH) once constituted the entire CHH family [25]. In our study, the number of DEGs decreased in the groups exposed to continuous light compared to those exposed to continuous darkness with an increasing number of days. DEG analysis implicated that juvenile hormone (JH)-inducible protein was downregulated (day 2), and CHH, insulin-like growth factor-binding protein, and gonadotropin-inducible transcription were upregulated (day 4). These differences may have been caused by short periods of light that cause environmental stresses on crabs (Table 2). In a study of environmental stress on Pacific white shrimp, DEGs that respond to environmental stress were identified, some of which included *JH protein* [26]. JH, which is similar to MOIH in structure and function, functions as an inhibitor of larval metamorphosis [27]. For some female insects, stress induced by photoperiods determine whether they enter reproductive stasis by affecting JH synthesis [28]. Eyestalk ablation promotes gonadal maturation and reproduction in female shrimp, mainly due to interference with the synthesis and release of GIH. CHH is a multifunctional endocrine hormone that is associated with physiological homeostasis and stress adaptation. CHH in the eyestalk not only regulates blood glucose but also synergizes with MIH to inhibit ecdysone secretion, which is widely expressed in the central and peripheral nervous system, including the blood, muscle, and hepatopancreas [29]. The broad tissue expression of CHH clearly indicates its functional importance and suggests that it might be closely related to that observed in other hormone genes.

GO analysis showed that genes were enriched in the purine-containing compound catabolic process, KEGG was enriched in phagosomes and metabolic pathways, and six genes were upregulated in the TCA pathway; thus, crab adaptation to photoperiod changes may require energy. Our findings are consistent with the results of a previous study on light damage in crustacean eyes, which found that light absorption and phototransduction were associated with the release of large amounts of energy [30]. MIH 1, crustacean female hormone, gonadotropin-releasing hormone receptor, CHH, and eclosion hormone did not display significant differential expression between the continuous light and continuous dark groups when compared with the normal group (days 2, 4, and 6) in the present study; however, differences in response time may exist. The slow response observed in the previous study is consistent with the temporal changes in CHH observed during the normal molt cycles of many crustacean species. In the molting process, the level of Pt-CHH1 in the eyestalk increased postmolt, was significantly higher at intermolt, and subsequently decreased during premolt. During ovarian development, the level of Pt-CHH1 in the eyestalk decreases from the previtellogenic stage and is significantly lower in the mature stage [31]. The process of light-induced hormone regulation suggests the existence of a relatively slow response-regulatory system. Similar conclusions were drawn in an environmental stress study in which crabs exposed to stressful conditions for 3 days showed significant differences in hemolymph ecdysteroid levels, which continued to decrease to 20% of those of the controls by day 14. Ecdysteroid titers of stressed crabs returned to prestress levels 7 days after stress termination [32]. The length of our experiment (6 days) was insufficient, and we will gradually improve the experimental design by extending the photoperiod to explore the CHH superfamily.

Humoral immune factors, including the anti-lipopolysaccharide factor, cytochrome P450, lectin, cathepsin H, and catalase, play crucial roles in the immune responses of crustaceans. Humoral immune factors are mainly effectors of blood lymphocytes with antibacterial, antiviral, bacteriolytic, recognition, and agglutination activities. Further, humoral immune factors recognize foreign substances and regulate and synergize the cellular immunity of blood lymphocytes to suppress and destroy pathogens [33]. As shown in Table 2, *the anti-lipopolysaccharide factor* is downregulated on days 2 and 4, while *cathepsin H* and *alpha-tubulin* is upregulated on days 4 and 6. Through KEGG enrichment analysis, we found that lectin, cathepsin H, and alpha-tubulin act on the phagosome pathway, and catalase is enriched in the MAPK pathway (Figure S6). Phagocytosis—a metabolic activity triggered by the binding of various receptors—is an important pathway for maintaining cellular homeostasis [34]. Therefore, lectin, cathepsin H, and alpha-tubulin may be involved in the maintenance of body homeostasis in crabs. 

Tubulin plays an indispensable role in maintaining cell shape, movement, and intracellular material transport [35]. Lectins are sugar-binding proteins recognized by C-type lectin receptors as sequences of glycolipids (or glycoproteins) on the surface of pathogenic cells [36]. When C-type lectin receptors bind, they activate macrophages and produce cytokines that can firmly bind to pathogens [37]. C-type lectin receptors capture and transport pathogens through the endocytic pathway. Cathepsin is the main protease in lysosomes and helps maintain cellular homeostasis. Under stress caused by changes in the light environment, lysosomes rupture and release cathepsin L and cathepsin H, which act via the phagocytic pathway. Lipopolysaccharide (LPS) is an antimicrobial peptide (AMP). A previous study found that a C-type lectin participated in the homeostatic regulation of hemolymph microbiota by maintaining AMP expression [38]. 

Currently, the MAPK pathway is known to play an important role in the immunity of crustaceans and is involved in regulating various processes such as cell proliferation, differentiation, apoptosis, and the inflammatory response. Transforming growth factor β-activated kinase 1 (TAK1) of shrimp conferred antibacterial protection through regulating the activation of the MAPK and NF-κB pathways [39]. However, research in vertebrates has identified a connection between the MAPK pathway and circadian rhythms. 

In 1998, light-evoked MAPK signaling tracks with clock-resetting effects of light were demonstrated in mouse experiments [40]. Our data indicated that although the MAPK signaling pathway was the only signaling pathway in the continuous light group, this pathway was absent in the continuous darkness group. Thus, we suggest that the MAPK pathway is highly responsive to light input during the subjective night and insensitive to light during the middle of the subjective day, which may explain why there were more DEGs exhibited in the continuous light group than in the continuous darkness group. Our findings are consistent with previous research results, indicating that light-evoked gene expression is limited to the nighttime domain; early-night light predominately leads to increased gene expression, and late-night light predominately leads to gene downregulation [41]. Within the suprachiasmatic nucleus (SCN), photic stimulation is coupled to clock entrainment via a number of intracellular signaling pathways, including nitric oxide/protein kinase G (PKG), calcium/calmodulin-dependent kinase II (CaMKII), and the ERK/MAPK pathway [40,42]. Signaling via the MAPK pathway shapes the response of the circadian clock to light [43]. Current research on the MAPK pathway has focused on its role in crustacean immunity. However, our data suggest that the mechanisms underlying the regulation of circadian rhythms via the MAPK pathway merit further investigation.

## 5. Conclusions

Our study provides comprehensive insights into the molecular adaptations of Chinese mitten crabs to diverse photoperiods, shedding light on the intricate pathways and genes involved in these processes. While our findings contribute to the understanding of how crustaceans respond to environmental changes at the molecular level, it is crucial to emphasize that our focus was primarily on molecular aspects rather than on physiological adaptations. Identification of the MAPK pathway as a potential regulator of circadian rhythms is a promising lead, suggesting avenues for further research. This study encourages future exploration, particularly with respect to unraveling the implications of the MAPK pathway in crustacean immunity and its role in coordinating responses to varying light conditions.

## Figures and Tables

**Table 1:** The primers sequence for real-time PCR.

Target Gene	Forward (5′–3′)	Reverse (5′–3′)
*β-actin*	GCCAAGACTCAACCCACGTAAA	TCACCGGCAAAACCAGACTT
*anti-lipopolysaccharide-factor 3*	GACCCTTTGCTGAATGCTTGA	CTGCTCTACAATGTCGCCTGA
*cathepsin L1-like*	ACAGGAAGCCGTTCATAGC	GCACGGTATGGGAAGCA
*cathepsin H*	CATTACGGAGAGAGGAAGCTTGT	GTTTTCCAGGAAGCACAAACTC
*crustacean hyperglycemic hormone*	GCTACAGCAACCTCGTCTTCCG	TTCTTCCTGCCAACCACCC
*c-type lectin*	ATGGGTGGAAGCGGTAGCC	TCGGGTGCCAGAAGGGAAT
*cytochrome P450*	CCAACTGTCTTGTTCTCCCCACT	CTCTTCTGCCGAGCATGTCTCA

**Table 2:** Up- and downregulation of DEG in E. sinensis.

Sample	Gene	Up/Down	*p* Value
EN2 vs. ED2	*cuticle protein 7-like*	up	0.001756418
	*cytochrome P450*	up	0.009890736
	*alpha-tubulin*	up	0.0220984
EN2 vs. EL2	*anti-lipopolysaccharide factor 3*	down	0.00011537
	*integrin alpha 4*	down	0.000588365
	*c-type lectin*	down	0.009270203
ED2 vs. EL2	*cathepsin L1-like*	up	0.000136118
	*c-type lectin*	up	0.015881644
	*juvenile hormone-inducible protein*	down	0.007739685
EN4 vs. ED4	*cytochrome P450*	down	6.48881 × 10^−7^
	*anti-lipopolysaccharide factor 2*	down	0.002221855
	*cuticle protein*	up	0.003159027
	*insulin-like growth factor-binding protein*	up	0.024775594
	*cathepsin L1-like*	up	0.031447548
EN4 vs. EL4	*alpha-tubulin*	up	2.55705 × 10^−6^
	*catalase*	up	0.010462105
	*gonadotropin inducible transcription*	up	0.02381274
	*cathepsin H*	up	0.015726694
	*anti-lipopolysaccharide factor*	down	0.002775506
	*anti-lipopolysaccharide factor 2*	down	0.011498019
ED4 vs. EL4	*cathepsin H*	up	6.89838 × 10^−6^
	*cytochrome P450 CYP2*	up	2.77952 × 10^−5^
	*cathepsin L1-like*	up	0.00432067
	*crustacean hyperglycemic hormone*	up	0.014342243
EN6 vs. ED6	*perlucin 5*	up	0.007388809
	*cytochrome P450*	up	0.014066799
EN6 vs. EL6	*alpha-tubulin*	up	9.17 × 10^−6^
	*heat shock 70 kDa protein*	up	1.68497 × 10^−5^
	*cathepsin H*	up	0.000438936
	*cathepsin L1-like*	up	0.00108408
	*catalase*	up	0.010191505
ED6 vs. EL6	*cathepsin H*	up	0.004655283
	*crustin 4*	down	0.02655283

**Table 3:** Hormone-related genes of *E. sinensis*.

Gene	Chromosome	Length	*p* Value
*crustacean hyperglycemic hormone*	chr50	339	0.096884486
*molt-inhibiting hormone 1*	chr38	513	0.264026133
*crustacean female hormone*	chr40	702	0.095992495
*gonadotropin-releasing* *hormone receptor*	chr3	633	0.134805803
*eclosion hormone*	chr33	264	0.572562442

## Data Availability

The raw reads have been deposited in the NCBI database (BioSample number SAMN38735307).

## Supplementary Materials

The following supporting information can be downloaded at: https://www.mdpi.com/article/10.3390/life14020209/s1, Figure S1: Gene Ontology (GO) classification showing DEGs for *Eriocheir sinensis* eyestalk in EN4 vs. ED4 (A), EN4 vs. EL4 (B); Figure S2: Gene Ontology (GO) classification showing DEGs for *Eriocheir sinensis* eyestalk in EN6 vs. ED6 (A), EN6 vs. EL6 (B), ED6 vs. EL6 (C); Figure S3: Classification statistics of KEGG metabolic pathway showing DEGs for *Eriocheir sinensis* eyestalk in EN2 vs. ED2 (A), ED2 vs. EL2 (B); Figure S4: Classification statistics of KEGG metabolic pathway showing DEGs for *Eriocheir sinensis* eyestalk in EN4 vs. ED4 (A), EN4 vs. EL4 (B), ED4 vs. EL4 (C); Figure S5: Localization of differentially expressed genes in the citrate cycle pathway. Upregulated genes are marked by red rectangles; Figure S6: Localization of differentially expressed genes in the phagosome pathway. Upregulated genes are marked by red rectangles; Figure S7: Classification statistics of KEGG metabolic pathway showing DEGs for *Eriocheir sinensis* eyestalk in EN6 vs. ED6 (A), EN6 vs. EL6 (B), ED6 vs. EL6 (C); Table S1: The summary information of transcriptomic sequences; Table S2: Evaluation of RNA-Seq Data of *E. sinensis*; Table S3: Reference genomic information of *E. sinensis*.
