# Supplementary material for: Impact Analysis of Photoperiodic Disorder on the Eyestalk of Chinese Mitten Crab (Eriocheir sinensis) through High-Throughput Sequencing Technology
Source: Life (Basel). 2024 Jan 31;14(2):209. doi: 10.3390/life14020209 (PMC10890049; doi:10.3390/life14020209)
Supplement: Supplementary file 1 [file life-14-00209-s001.zip › Supplemental Table S1.pdf]

Supplemental Table S1. The summary information of transcriptomic sequences

Sample	Reads No.	Bases (bp)	Q20 (%)	Q30 (%)
2-E-L-1	47,531,556	7,129,733,400	96.70	92.12
2-E-L-2	51,086,712	7,663,006,800	96.41	91.57
2-E-L-3	48,875,700	7,331,355,000	95.90	90.64
2-E-D-1	45,190,922	6,778,638,300	96.65	92.00
2-E-D-2	45,148,566	6,772,284,900	96.53	91.88
2-E-D-3	49,970,130	7,495,519,500	97.02	92.66
2-E-N-1	49,819,870	7,472,980,500	96.63	91.95
2-E-N-2	46,510,268	6,976,540,200	96.75	92.08
2-E-N-3	40,975,142	6,146,271,300	96.71	92.05
4-E-L-1	48,486,932	7,273,039,800	96.50	91.64
4-E-L-2	46,297,908	6,944,686,200	96.61	91.92
4-E-L-3	44,600,702	6,690,105,300	96.63	91.91
4-E-D-1	50,772,066	7,615,809,900	96.79	92.21
4-E-D-2	44,818,322	6,722,748,300	96.44	91.63
4-E-D-3	52,678,496	7,901,774,400	96.77	92.31
4-E-N-1	43,086,798	6,463,019,700	96.82	92.35
4-E-N-2	45,452,204	6,817,830,600	96.79	92.22
4-E-N-3	46,520,634	6,978,095,100	96.60	91.89
6-E-L-1	43,720,684	6,558,102,600	96.20	91.18
6-E-L-2	40,473,144	6,070,971,600	96.39	91.46
6-E-L-3	45,273,738	6,791,060,700	96.69	92.01
6-E-D-1	45,794,100	6,869,115,000	96.08	90.89
6-E-D-2	42,571,902	6,385,785,300	96.51	91.66
6-E-D-3	45,602,772	6,840,415,800	96.34	91.49
6-E-N-1	43,973,142	6,595,971,300	96.67	92.06
6-E-N-2	46,805,456	7,020,818,400	96.58	91.95
6-E-N-3	49,358,704	7,403,805,600	96.25	91.34
