# Supplementary material for: Impact Analysis of Photoperiodic Disorder on the Eyestalk of Chinese Mitten Crab (Eriocheir sinensis) through High-Throughput Sequencing Technology
Source: Life (Basel). 2024 Jan 31;14(2):209. doi: 10.3390/life14020209 (PMC10890049; doi:10.3390/life14020209)
Supplement: Supplementary file 1 [file life-14-00209-s001.zip › Supplemental Table S2.pdf]

Supplemental Table S2. Evaluation of RNA-Seq Data of *E. sinensis*

Sample	Clean Reads No.	Clean Data (bp)	Clean Reads %	Clean Data %
2-E-L-1	42,645,506	6,396,825,900.00	89.72	89.72
2-E-L-2	45,663,482	6,849,522,300.00	89.38	89.38
2-E-L-3	43,773,492	6,566,023,800.00	89.56	89.56
2-E-D-1	41,342,194	6,201,329,100.00	91.48	91.48
2-E-D-2	40,255,534	6,038,330,100.00	89.16	89.16
2-E-D-3	44,907,644	6,736,146,600.00	89.86	89.86
2-E-N-1	44,640,118	6,696,017,700.00	89.60	89.60
2-E-N-2	41,815,266	6,272,289,900.00	89.90	89.90
2-E-N-3	36,867,796	5,530,169,400.00	89.97	89.97
4-E-L-1	43,129,466	6,469,419,900.00	88.95	88.95
4-E-L-2	41,372,870	6,205,930,500.00	89.36	89.36
4-E-L-3	40,537,772	6,080,665,800.00	90.89	90.89
4-E-D-1	45,863,414	6,879,512,100.00	90.33	90.33
4-E-D-2	40,072,550	6,010,882,500.00	89.41	89.41
4-E-D-3	40,259,978	6,038,996,700.00	76.42	76.42
4-E-N-1	37,907,576	5,686,136,400.00	87.97	87.97

4-E-N-2	40,516,880	6,077,532,000.00	89.14	89.14
4-E-N-3	41,710,084	6,256,512,600.00	89.65	89.65
6-E-L-1	38,398,750	5,759,812,500.00	87.82	87.82
6-E-L-2	36,075,812	5,411,371,800.00	89.13	89.13
6-E-L-3	35,718,558	5,357,783,700.00	78.89	78.89
6-E-D-1	40,708,160	6,106,224,000.00	88.89	88.89
6-E-D-2	38,666,090	5,799,913,500.00	90.82	90.82
6-E-D-3	41,046,054	6,156,908,100.00	90.00	90.00
6-E-N-1	39,496,764	5,924,514,600.00	89.82	89.82
6-E-N-2	42,030,634	6,304,595,100.00	89.79	89.79
6-E-N-3	44,162,904	6,624,435,600.00	89.47	89.47
