# Supplementary material for: Impact Analysis of Photoperiodic Disorder on the Eyestalk of Chinese Mitten Crab (Eriocheir sinensis) through High-Throughput Sequencing Technology
Source: Life (Basel). 2024 Jan 31;14(2):209. doi: 10.3390/life14020209 (PMC10890049; doi:10.3390/life14020209)
Supplement: Supplementary file 1 [file life-14-00209-s001.zip › Supplemental Table S3.pdf]

Supplemental Table S3. Reference genomic information of *E. sinensis*

**Database**	**Number**	**Percentage**
NR	78701	41.62
GO	50358	26.63
KEGG	36889	19.51
eggNOG	67040	35.46
Swissprot	51563	27.27
In all database	32406	17.14
