# Supplementary figures and images for: Impact Analysis of Photoperiodic Disorder on the Eyestalk of Chinese Mitten Crab (Eriocheir sinensis) through High-Throughput Sequencing Technology
Source: Life (Basel). 2024 Jan 31;14(2):209. doi: 10.3390/life14020209 (PMC10890049; doi:10.3390/life14020209)

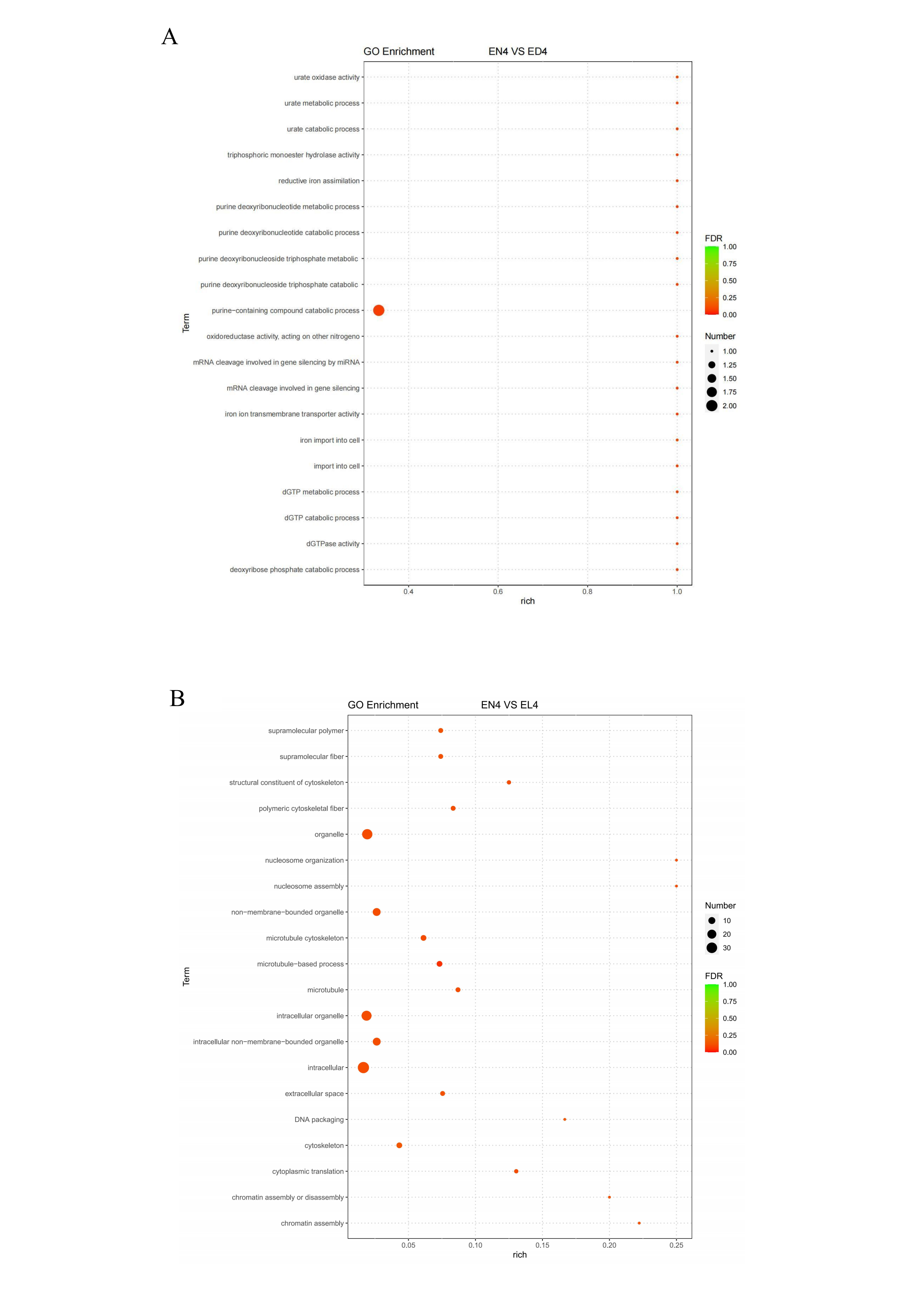

Supplement: Supplementary file 1 [file life-14-00209-s001.zip › Figure S1.tif]

A

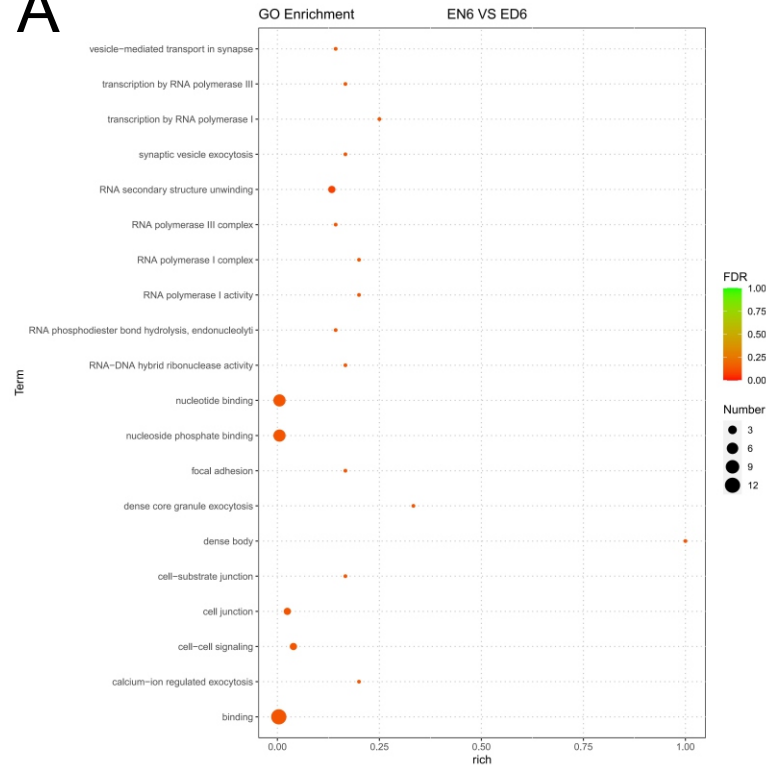

B

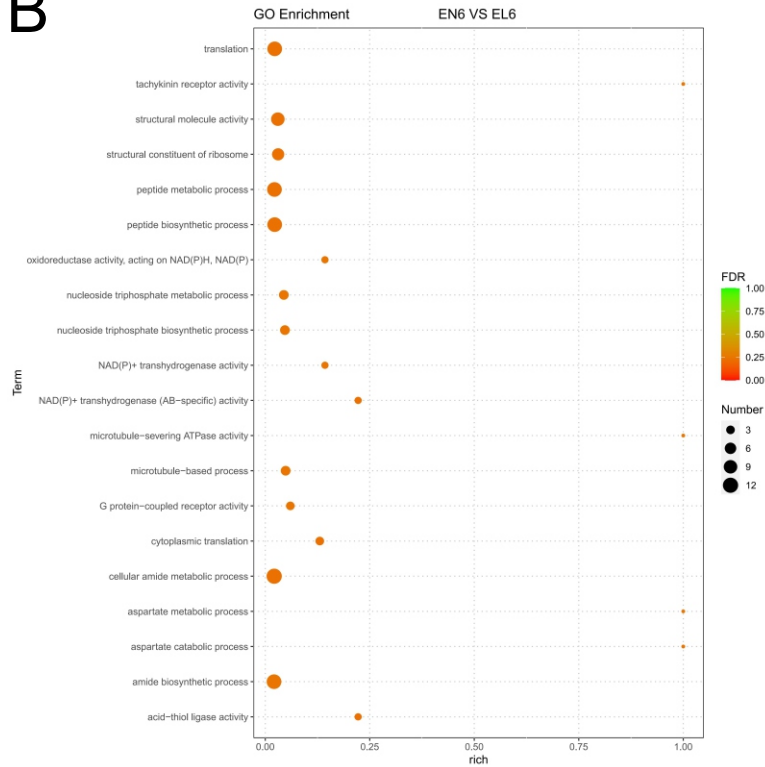

C

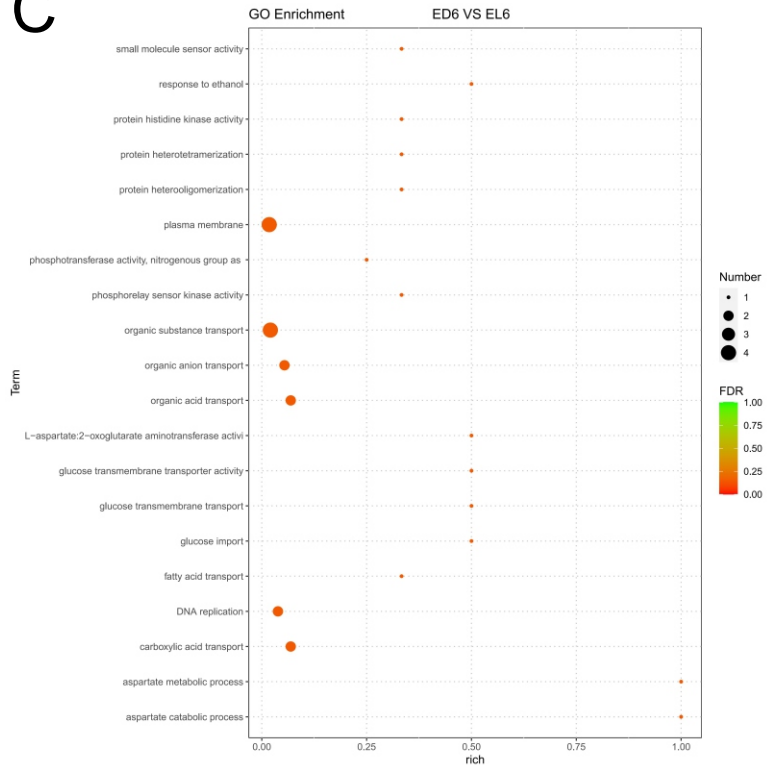

Supplement: Supplementary file 1 [file life-14-00209-s001.zip › Figure S2.pdf]

A

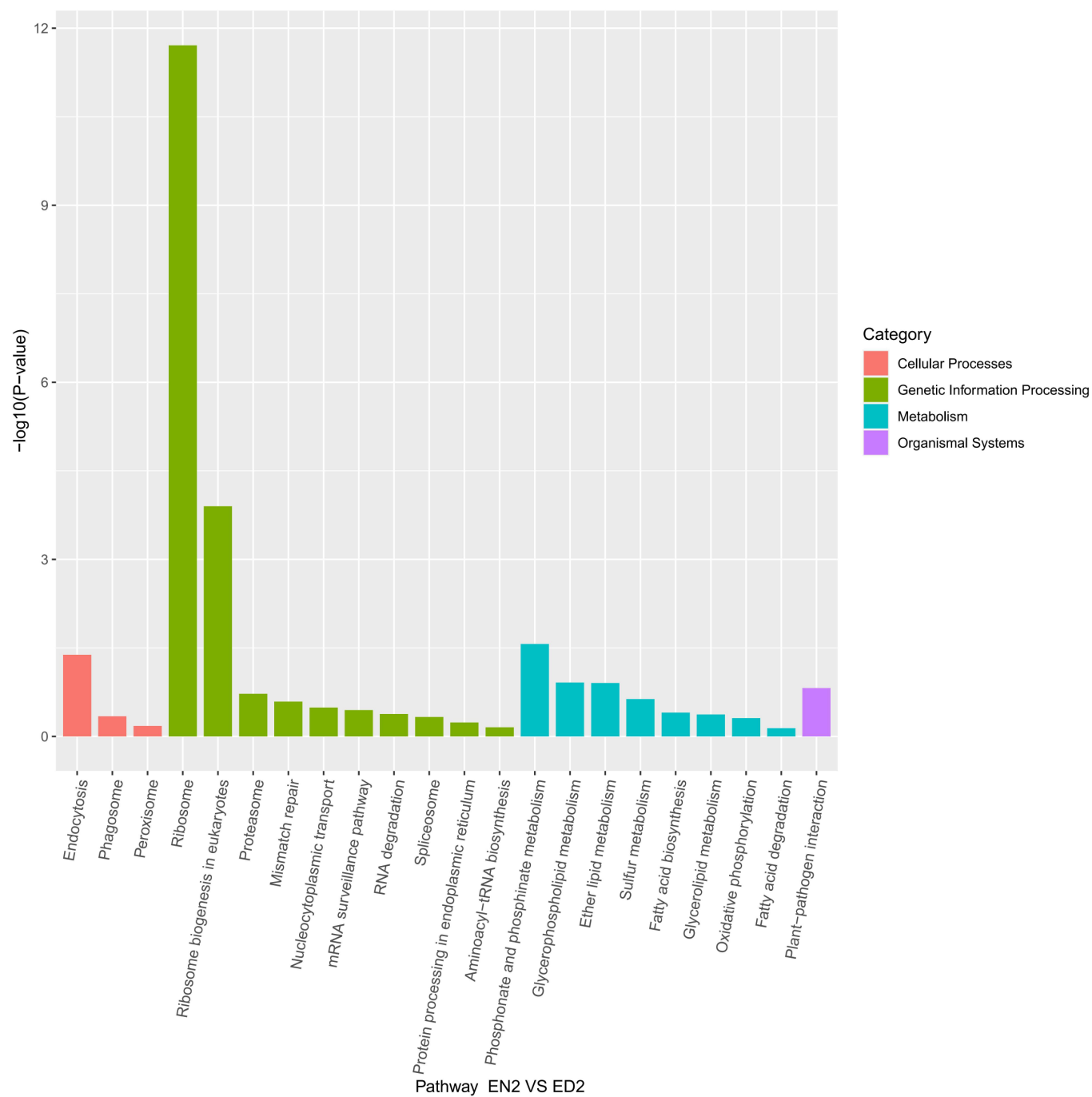

B

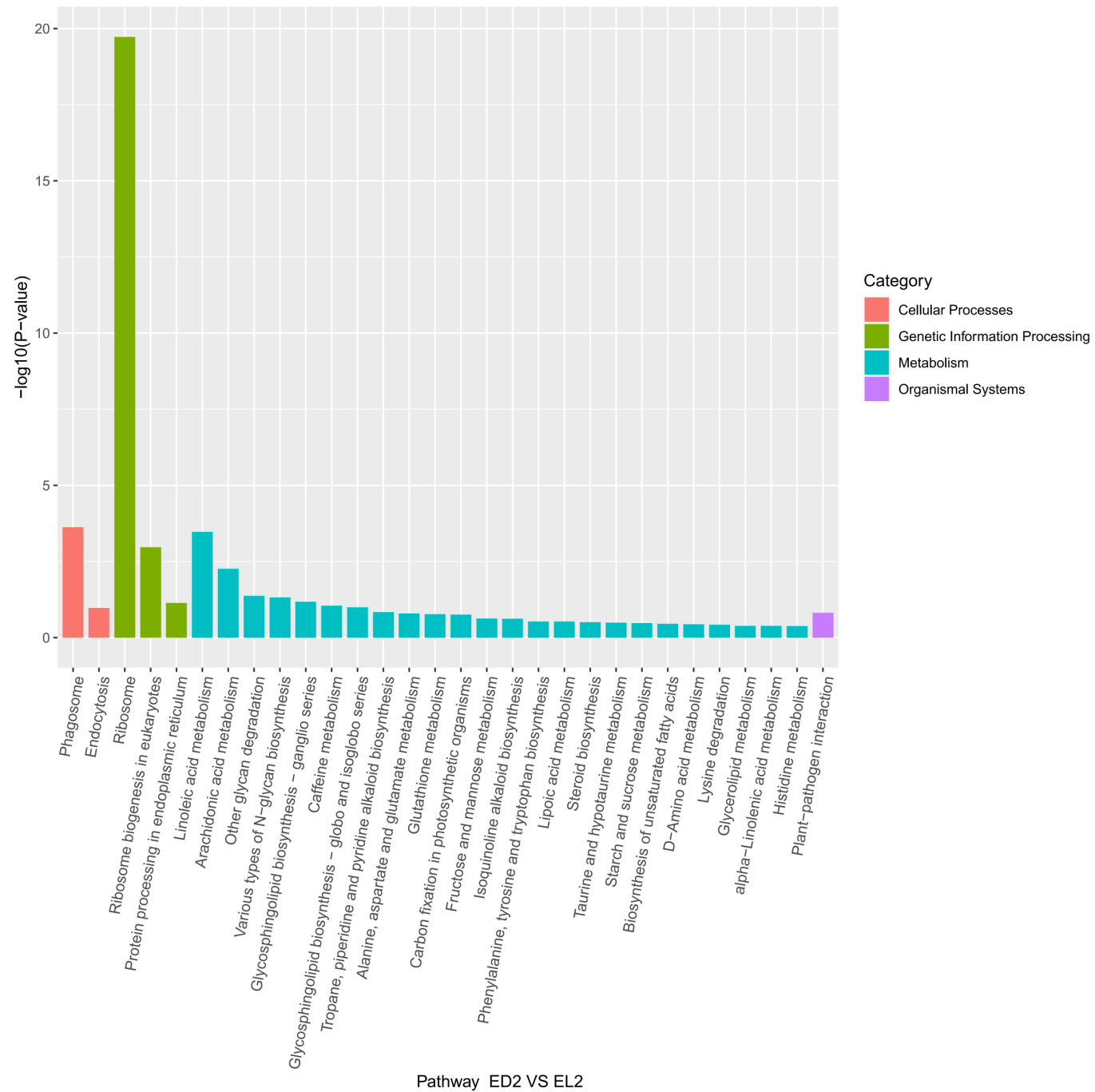

Supplement: Supplementary file 1 [file life-14-00209-s001.zip › Figure S3.pdf]

A

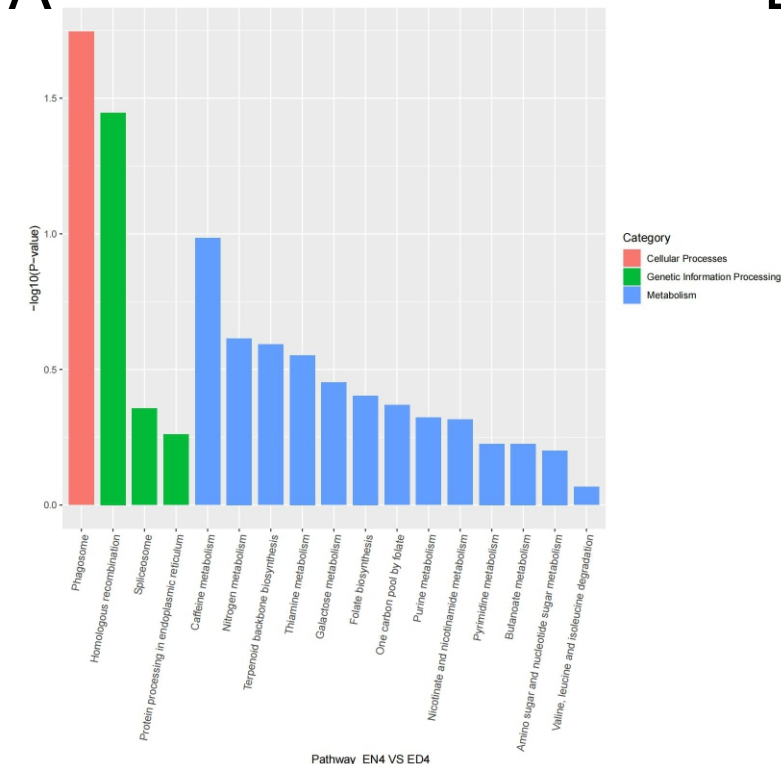

B

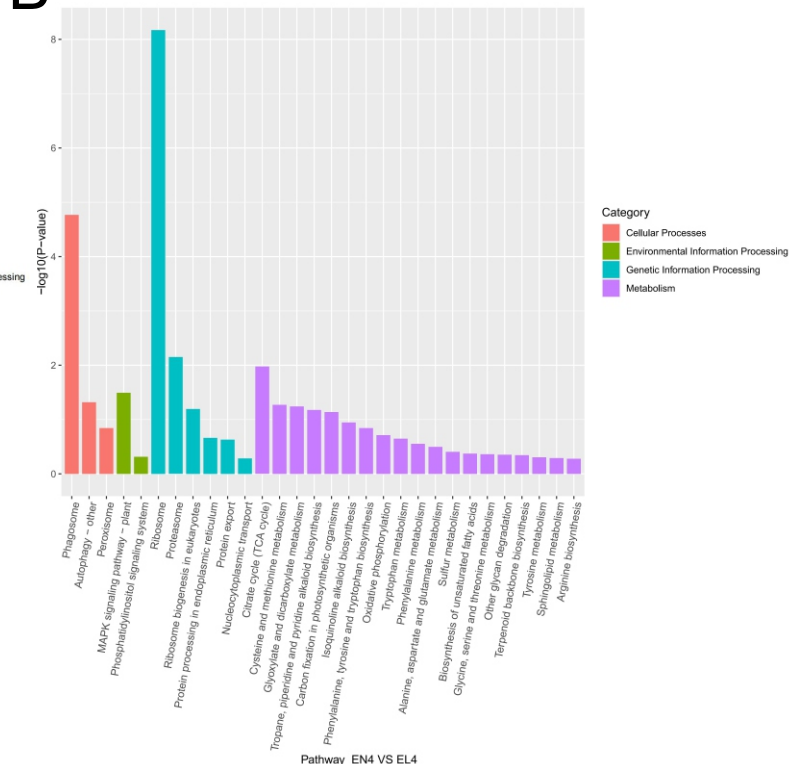

C

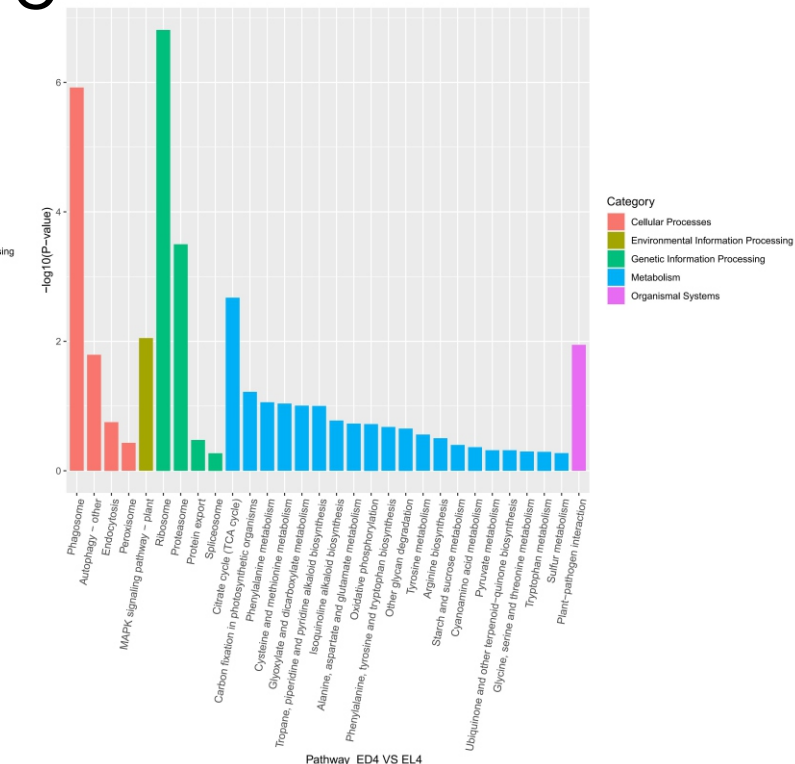

Supplement: Supplementary file 1 [file life-14-00209-s001.zip › Figure S4.pdf]

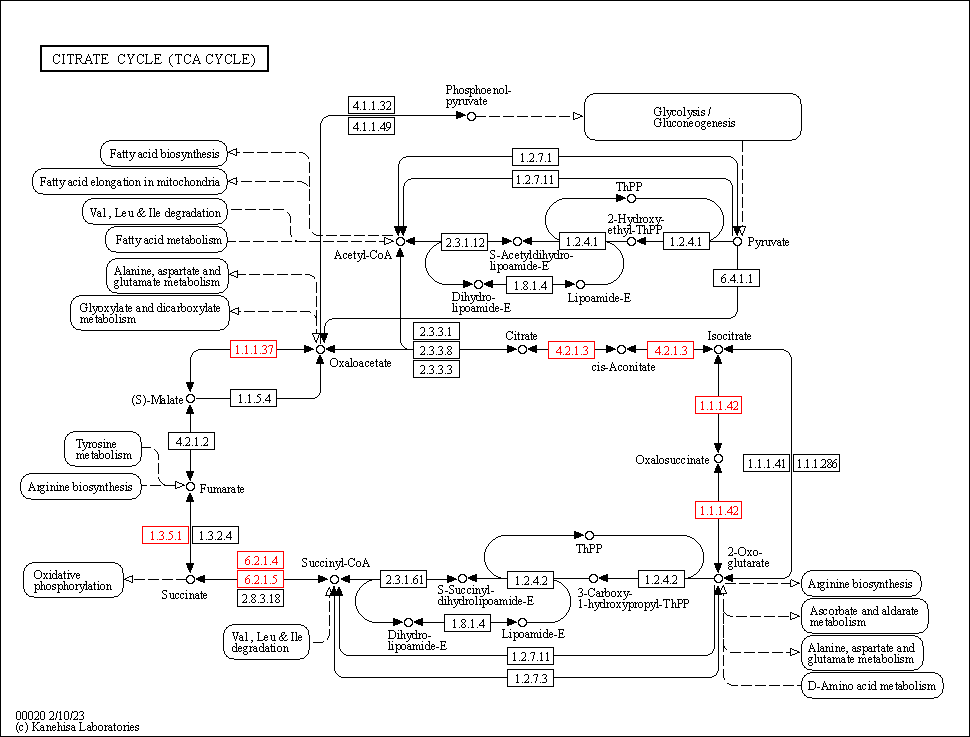

Supplement: Supplementary file 1 [file life-14-00209-s001.zip › Figure S5.png]

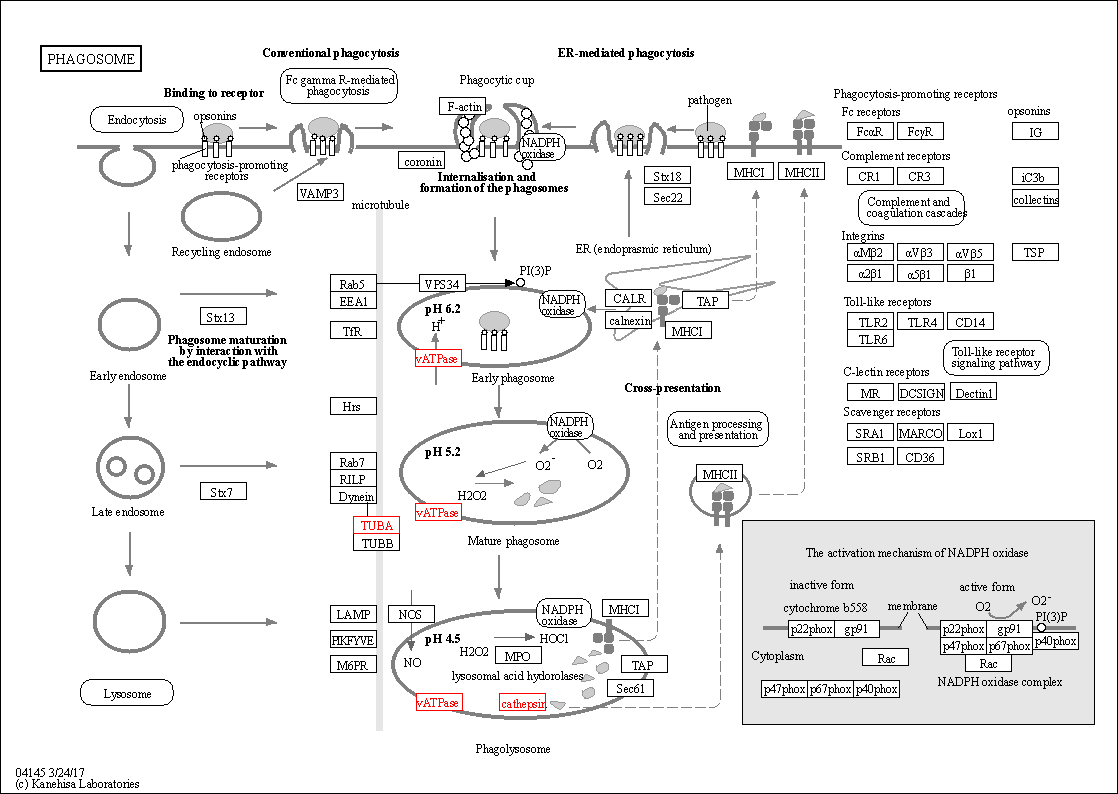

Supplement: Supplementary file 1 [file life-14-00209-s001.zip › Figure S6.png]

C

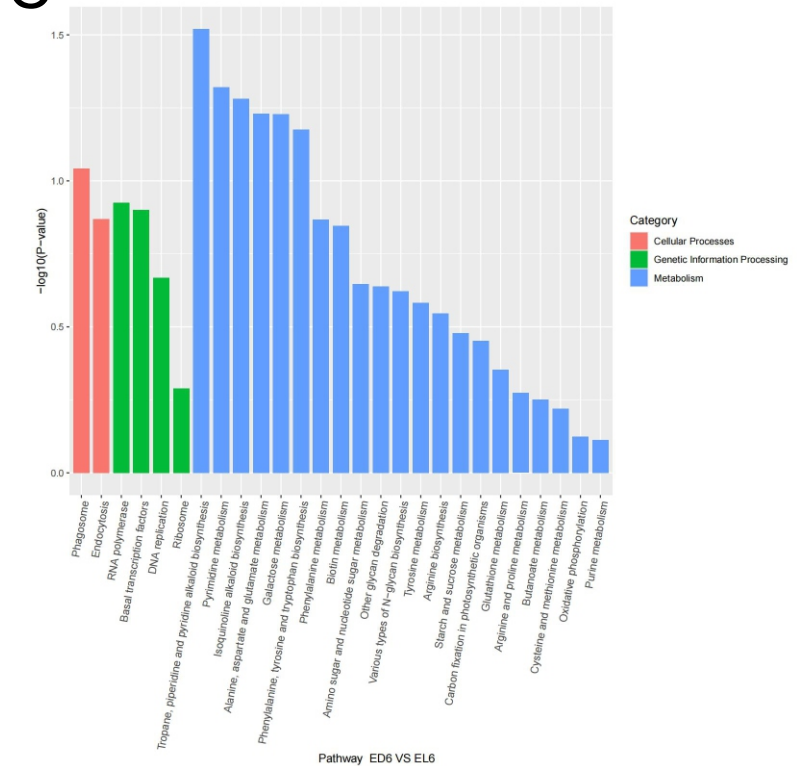

Supplement: Supplementary file 1 [file life-14-00209-s001.zip › Figure S7.pdf]
